# Over-Indebtedness and Problem Gambling in a General Population Sample of Online Gamblers

**DOI:** 10.3389/fpsyt.2020.00007

**Published:** 2020-02-04

**Authors:** Anders Håkansson, Carolina Widinghoff

**Affiliations:** ^1^Department of Clinical Sciences Lund, Psychiatry, Faculty of Medicine, Lund University, Lund, Sweden; ^2^Gambling disorder unit, Malmö Addiction Center, Malmö, Sweden

**Keywords:** gambling disorder, problem gambling, indebtedness, addiction, online gambling

## Abstract

**Background:**

Online gambling has increased in recent years, including online casino games and live sports betting which constitute rapid gambling activities with significant potential for gambling-related harm. There is a paucity of research examining whether specific gambling patterns are related to problem gambling and over-indebtedness, when controlling for psychological distress, gender, and other risk factors.

**Methods:**

A general population-based web panel of 1,004 online gamblers were examined in an online survey addressing problem gambling symptoms (the PGSI), psychological distress (Kessler- 6), past 30-day gambling activities, past 30-day gambling losses, history of subjective over-indebtedness and expected over-indebtedness in the near future, as well as socio-demographic data.

**Results:**

In logistic regression analyses, problem gambling was associated with psychological distress, recent online casino gambling, and recent combined online casino gambling and live sports betting. History of over-indebtedness was associated with recent combined online casino gambling and live sports betting, and expected over-indebtedness was associated with online casino gambling. Problem gambling, and a history of having borrowed money for gambling, were markedly higher in online casino gamblers, compared to subjects not reporting this gambling activity. Problem gambling was markedly more common in women, but was not associated with gender in the adjusted analysis.

**Conclusions:**

In online gamblers, high rates of problem gambling and over-indebtedness were seen, and online casino gambling (alone or in combination with live sports betting), was associated with remarkably increased risk. Gender distribution of problem gamblers was clearly in contrast to that found in previous problem gambling literature. These findingsa suggest regulations in the market of online casino gambling, and prevention of over-indebtedness in gambling-related borrowing, in consumer credit counselling and in mental health care. In particular, female gender may need to be addressed as a stronger risk factor than previously described.

## Background

Gambling disorder is a psychiatric condition listed among addictive disorders along with alcohol and drug use disorders (1), affecting around 0.5% of the general adult population (2–4). A higher number of individuals are believed to suffer from problem gambling, a problematic gambling pattern which may or may not fulfil diagnostic criteria. Prevalence of problem gambling varies across studies and settings, from below 1% of the adult population, up to around 5% to 6% (5). Gambling-related problems may have severe consequences for the individual, including financial debts (6), suicidal ideation, suicide attempts (7), and an increased risk of completed suicide (8). A recent study demonstrated that problem gambling may lead to psychological distress both through a direct pathway, and through a pathway mediated *via* financial debts (9), demonstrating the important role of debts in the problem pattern described in patients with a gambling disorder. Financial debts contribute to psychological distress and suicidal behavior independent of gambling disorder (10, 11). Over-indebtedness, which refers to an incapacity to fulfil payback requirements, is associated with depressive symptoms, suicidal ideation (12), and sleeping problems (13).

The past few years have seen an increase in the proportion of online gambling in populations seeking treatment for a gambling disorder. Importantly, authors have argued that online gambling may be particularly addictive (14), due to its speed and availability (15). Also, others have indicated that even though the literature is limited, people gambling online may be particularly likely to have comorbid substance use or mental health problems (16). Meanwhile, the opposite finding of lower psychological distress in online gamblers compared to land-based gamblers, also has been demonstrated. However, there is evidence that treatment uptake may be lower in online gamblers, theoretically contributing to the assumption of a more problematic pattern in online gambling (17).

In Sweden, the setting addressed in the present study, several signs indicate an increase in online gambling in recent years. As many as 89% of treatment-seeking patients with gambling disorder reported an online gambling activity to constitute their problematic gambling (18). Meanwhile, governmental sources in Sweden have reported that foreign-based online gambling operators were gaining larger and larger shares of the gambling market, typically providing online casino gambling and online sports betting. Also, in helpline callers in Sweden, the share of problem gamblers reporting online gambling has increased in recent years, with online casino being by far the most common among these (19). Along with concerns raised about online casino gambling, authors have also highlighted the addictive potential of live sports betting, i.e. different types of “in-play” betting during sports events (20). In particular, in-play or live betting has been shown to be associated with a higher degree of problem gambling among sports bettors (21, 22); a recent study reported that among sports bettors, problem gambling was seen in as many as 79% of those betting on in-play micro events, compared to only 29% of other bettors (23).

Thus, increasing concerns about online gambling are raised due to increasing proportions of online gamblers among treatment-seeking patients. In Sweden, a large majority of gambling advertisements in television have been reported to represent online gambling, where the gambling advertisements promoting online casino by far outnumber those representing other gambling activities (24). At the same time, changing gender patterns have been described in the most recent general population survey of the national Institute of Public Health, and based on this survey, media reported that the proportion with the highest degree of problem gambling in the survey were more likely to be women than men (25). As this gender distribution is in contrast with previous literature, more research addressing gender differences in novel gambling patterns is warranted (18, 19).

In relation to the increase in online gambling, and the theoretically enhanced risk of debts developing rapidly in the online gambling modality, there is need for more research examining how different online gambling activities are related to problem gambling and over-indebtedness. Therefore, the present study aimed to examine financial debts and problem gambling symptoms in online gamblers, and how debts and problem gambling are related to psychological distress, specific online gambling activities, gender, and socio-demographic data. Based on the reporting of online gambling in patients seeking treatment, the study hypothesized that problem gambling would differ by past-month gambling activities reported. Also, based on the reporting of a possible increase in female problem gambling in Sweden in a recent public health report (25), the study hypothesized that male gender would not be as strong a predictor of problem gambling as in previous studies (18, 19). In addition, given the role of indebtedness in the harm related to gambling, the present study also hypothesized that over-indebtedness would differ by gambling activities reported.

## Methods

### Design

The present study is a cross-sectional survey study conducted in a sample derived from the adult general population, and recruited from a pre-existing web panel of a national market research company. The study was approved by the regional ethics board in Lund, Sweden (file number 2018/495).

### Participants

The aim of the study was to address roughly 1,000 adult individuals from the general population, stratified for age, who had gambled for money on either an online casino site or an online betting site on at least 10 occasions during the past 12 months. The participants were recruited from the web panel of the market survey company Ipsos, which typically addresses individuals in their web panel for commercial markets surveys and political opinion polls. Here, web panel members were asked the following question: “If you think about the past 12 months, how often have you gambled on betting services online or online casinos?” and they were given the following options to answer: “1–4 times,” “5–9 times,” “unsure/don’t know,” and “10 times or more.” Only individuals endorsing the latter option were further included in the study, and offered a written study information online. Clients were moved further to the survey only if they gave online written informed consent to the study. Finally, a total number of 1,009 individuals answered the survey.

Problem gambling was measured using the Problem Gambling Severity Index (PGSI, 26), and for this nine-item variable, answers were missing on any of the items for a total of 20 individuals. In order to minimize missing data, respondents who fulfilled criteria of at least a moderate-risk gambling, i.e. a PGSI score of at least three, from the items they responded to, were also included (indicating at least moderate-risk gambling, n = 15), and for these individuals, the value zero was imputed for missing items. Five individuals, however, had either no items at all (n = 2), only two items with a total score of zero (n = 1), or eight available items with a total score of less than two (n = 2). Thus, with a remaining uncertainty about whether these individuals were non-problem gamblers or had a moderate-risk or problem gambling, these five individuals were excluded, leaving a final sample of 1,004 included subjects, 78% of whom were men. Characteristics of the included sample are displayed in **Table 1**. Response time of the survey had a median of 363 seconds (inter-quartile range, 288–455.5; range, 115–1,750 sec).

**Table 1:** Sample characteristics (N = 1,004).

	n	%
Gender		
- Male	786	78
- Female	218	22
Age		
- 18–24 years	41	4
- 25–29 years	79	8
- 30–39 years	220	22
- 40–49 years	227	23
- 50–59 years	219	22
- 60–69 years	141	14
- 70 years and older	77	8
Living conditions		
- Alone with children	66	7
- Alone without children	231	23
- With partner and children	376	37
- With partner, without children	307	31
- With parents	24	2
Main occupation		
- Student	47	5
- Working	727	72
- Job seeking	29	3
- Retired	170	17
- Other	31	3
Highest level of education		
- Mandatory primary school	71	7
- High school	447	45
- University studies without full degree	154	15
- Full university degree	317	32
- Other	15	1
Daily tobacco smoker or “snuff” user		
- Yes	406	40
- No	592	59
- Wish not to answer	6	1
Ever prescribed pharmaceuticals or psychotherapy for psychological distress		
- Yes	214	21
- No	780	78
- Wish not to answer	10	1
Ever felt a need to seek treatment for problem gambling		
- Yes	60	6
- No	936	93
- Wish not to answer	8	1
Ever felt a need to seek treatment for alcohol problems		
- Yes	65	6
- No	929	93
- Wish not to answer	10	1
Ever felt a need to seek treatment for drug problems		
- Yes	34	3
- No	962	96
- Wish not to answer	8	1

### Measures

#### Gambling Involvement

Participants were asked about their gambling involvement across ten different gambling activities during the past 30 days. The gambling activities listed, for land-based and online gambling modalities, were based on those included in the Swedish updated version of the Addiction Severity Index-gambling (ASI-gambling), updated by the authors and available online from the National Board of Health and Welfare (27). The ASI-gambling is a Swedish adaptation of previously known versions of the ASI for the purpose of measuring gambling patterns and problem gambling (28). Here, the Swedish ASI gambling version served as a template for gambling activities to be included in the study. The options provided were the following; casino games online, land-based casino, online horse race betting, land-based horse race betting, sports and odds (live betting), sports and odds (non-live betting), online poker, land-based poker, land-based electronic gambling machines, and online bingo. Given the focus on online gambling in the present study and the predominance of online casino and sports betting in help-seeking patients in Sweden (18, 19), clients were *post hoc* divided into categories depending on their recent (30-day) gambling on online casino (referred to as “casino games on the internet”) and live sports betting (“sports and odds—live betting”). Based on the way this wording is typically used in gambling advertising in Sweden (24), online casino games are likely to be understood by the general public as slot games, although other casino games appearing online also could be understood by this term. The variables related to online casino gambling and live sports betting were categorized into the following; clients gambling on 1) both these two gambling activities, 2) online casino but not live sports betting, 3) live sports betting but not online casino gambling, or 4) none of these two gambling activities. The emphasis on these two gambling activities was based on the fact that they represent the two leading gambling activities among patients seeking gambling disorder treatment in Sweden (14). As a co-factor to control for, an individual’s overall gambling involvement was measured with a categorical question about the size of losses from gambling during the past 30 days. Gambling losses were reported in categories derived from a previous epidemiological study in Denmark, a country with socio-demographic characteristics comparable to those of Sweden, where gambling losses and beliefs about peer gambling losses were highly associated with problem gambling (29). Thus, the same categories (although in Swedish instead of Danish currency) were used.

#### Psychological Distress

Psychological distress was measured using the Kessler Scale-6, K-6 (30). This scale includes six core symptoms, and the respondent endorsed criteria based on six Likert scales describing how often the respondent has perceived each particular symptom. The symptoms included are nervousness, feelings of hopelessness, restlessness/fidgety, depression, feelings of being worthless, and the feeling that everything in life is an effort. The K-6 internationally has been shown to perform well as a measure of psychological distress [see for example (31–33)].

#### Problem Gambling

Problem gambling was measured using the Problem Gambling Severity Index, PGSI (26). This scale includes nine symptoms related to gambling problems, and constitutes a well- established measure for epidemiological studies of problem gambling in the general population (4, 34). Here, respondents were categorized as in previous use of the total score of the instrument, with a sum of eight or more describing problem gambling, 3–7 indicating moderate-risk gambling, 1–2 indicating low-risk gambling, and a sum of 0 representing no risk (4).

#### Financial Debts and Over-Indebtedness

Brief questions assessed monthly income, and history of loans, the latter also including questions about whether an individual had ever borrowed money in order to be able to gamble or in order to cover losses derived from gambling, and whether this had happened during the past 12 months. Also, the respondents were asked whether they had had debts which during the past 12 months had been passed on to a collection service or to the Swedish Enforcement Authority.

Over-indebtedness has been defined in different ways in previous literature, and definitions tend to fall into three categories; an administrative definition, an objective definition, and a subjective definition (12). The latter is based on the individual’s own reporting of being over-indebted, i.e. self-reporting an incapacity to fulfil financial obligations. The Swedish Enforcement Authority (35) in previous reports uses a subjective definition, and in the present study, we therefore assessed over-indebtedness as derived from the following questions; “have you experienced that you (or you and others living in your household) have repeatedly recurring problems paying your bills?” (divided into two questions addressing the past year and lifetime prior to that), and “do you expect that you (or you and others living in your household) will have large problems paying your bills during the next two months?” (addressing expected over-indebtedness during the next 2 months). These items were derived from the wording used in a previous report on over-indebtedness from the Swedish Enforcement Authority (34).

#### Socio-Demographic Data

In addition, other variables included in the analyses addressed gender and age group, whether an individual ever had felt a need to seek treatment for alcohol problems, or problems related to illicit drugs or pharmaceuticals with addiction potential, and the level of education was addressed, and categorized in the analyses as whether an individuals had any post-high-school education, or not.

### Statistical Analyses

In addition to descriptive data, analyses examined variables associated with three outcome measures, respectively; a history of over-indebtedness (ever), expected over-indebtedness during the next two months, and problem gambling. In the examination of factors associated with each outcome measure, logistic regression was used. Given the high correlation between PGSI values and recent types of online gambling in a bivariate correlation matrix run prior to logistic regression analyses (Pearson R = 0.43), the full analyses for over-indebtedness were chosen to be hierarchical logistic regression analyses in two steps. Here, the first model included both PGSI score and online gambling activities, whereas the PSGI score was omitted from the second model. In these regression models, variables were entered simultaneously, and calculated with respect to the odds ratio of each variables with a 95-percent confidence interval. The variables included were gender, age, tertiary education (any education beyond high school), problem gambling described as PGSI score (in model 1 only), psychological distress, need for help for alcohol problems, need for help for drug problems, total gambling losses during the past 30 days, and recent specific online gambling activities. The latter was expressed as a four-level categorical variable describing the occurrence of online casino gambling and online sports betting, respectively. In the logistic regression where problem gambling (defined by cut-offs in the PGSI) was used as the dependent variable, the same independent variables as above were entered. For all logistic regression models, the Nagelkerke value is reported.

For descriptive purposes, unadjusted comparisons were carried out using chi-square method for categorical variables and the Mann-Whitney test for the continuous variable (psychological distress, the summed Kessler-6 value, as this was assumed not to be equally distributed).

## Results

Sample characteristics are reported in **Table 1**, and past 30-day gambling activities are listed in **Table 2**. In the sub-division of the sample with respect to online casino gambling and live sports betting, respondents were categorized into those reporting both online casino and live betting (18%, n = 177), those reporting online casino but no live betting (16%, n = 164), those reporting live betting but no online casino (36%, n = 365), and those reporting neither online casino nor live sports betting during the past 30 days (30%, n = 298).

**Table 2:** Past 30-day gambling activities reported (N = 1,004).

	n	%
Online casino	341	34
Live sports betting	542	54
Online horse betting	400	40
Online poker	179	18
Online bingo	161	16
Sports betting—non-live	605	60
Land-based casino	90	9
Land-based horse betting	219	22
Land-based poker	93	9
Land-based electronic gaming machines	104	10
Gambling for money within video games	79	8

### Problem Gambling

Thirteen percent met criteria of problem gambling, and another 19% met criteria of moderate-risk gambling. Another 23% met criteria of low-risk gambling, whereas the remaining 44% had no gambling problem. Problem gambling was significantly more common in women (24%) than in men (10%, p < 0.001), and the total percentage of moderate-risk or problem gambling was 48% in women and 28% in men (p < 0.001). Six percent of respondents reported a history of feeling a need to seek treatment for problem gambling.

The percentage of respondents with problem gambling was 44% in subjects reporting both past 30-day online casino gambling and live betting, and 18% in those reporting online casino but no live betting. In subjects reporting only live betting and no casino gambling, as well as in subjects reported neither of these gambling activities, 4% were problem gamblers. The summed measure of moderate-risk gambling and problem gambling was met by 65% of subjects reporting both online casino and live betting, and by 52% of those reporting only online casino gambling during the past 30 days, whereas the same measure was met by 21% of subjects reporting only live betting and no past 30-day online casino gambling, and by 16% reporting neither of these gambling activities during the past 30 days.

The unadjusted comparison between problem gamblers and non-problem gamblers is displayed in **Table 3**. In logistic regression (**Table 3**), problem gambling was significantly associated with younger age, psychological distress, a higher level of education, level of recent gambling losses, and with recent online casino gambling and recent combined online casino gambling and live sports betting.

**Table 3:** Comparison of respondents with problem gambling and no problem gambling. Chi-squared analysis and Mann-Whitney comparison, and logistic regression analysis with problem gambling as the dependent variable (N = 1,004).

	Problem gambling, % (n)	No problem gambling, % (n)	p value (Chi-squared or Mann-Whitney comparisons)	Odds ratio with 95% confidence interval (logistic regression)*
Male gender	61 (80)	81 (706)	<0.001	0.64 (0.36–1.15)
Age group			<0.001	0.57 (0.46–0.70)**
- 18–24	13 (17)	3 (24)	
- 25–29	18 (24)	6 (55)		
- 30–39	13 (41)	21 (179)		
- 40–49	22 (29)	23 (198)		
- 50–59	13 (17)	23 (202)		
- 60–69	2 (3)	16 (138)		
- 70+	1 (1)	9 (76)		
Psychological distress	16.5 (IQR 13–20)	9 (7–12)	<0.001	1.15 (1.09–1.21)**
Alcohol problems	18 (24)	5 (41)	<0.001	1.53 (0.64–3.64)
Drug problems	14 (19)	2 (15)	<0.001	2.40 (0.86–6.70)
Tertiary education	57 (75)	45 (396)	0.01	1.68 (1.01–2.80)**
Past 30-day money lost from gambling (Euros)***			<0.001	1.58 (1.41–1.78)**
- <4.3	5 (7)	21 (179)		
- 4.3–8.7	4 (5)	11 (94)		
- 8.7–17.4	5 (7)	16 (142)		
- 17.4–34.8	8 (10)	18 (156)		
- 34.8–52.2	15 (20)	13 (111)		
- 52.2–87.0	10 (13)	9 (77)		
- 87.0–173.9	18 (24)	9 (78)		
- 173.9–434.8	12 (16)	3 (26)		
- 434.8–869.6	8 (10)	1 (7)		
- >869.6	15 (20)	0 (2)		
Past-30-day online gambling activity			<0.001	
- None	8 (11)	33 (287)		2.40 (1.03–5.58)**
- Online casino, no live betting	23 (30)	15 (134)		
- Live betting, no online casino	11 (14)	40 (351)		0.74 (0.31–1.80)
- Both	58 (77)	11 (100)		5.12 (2.35–11.17)**

*Nagelkerke 0.56.

**Significant association (p < 0.05).

***Responses in local currency, and values expressed here in Euros (1 Euro corresponding to around 11.5 SEK).

### Lifetime History of Over-Indebtedness

The item describing a lifetime history of over-indebtedness was endorsed by 10%, whereas the following item, specifying whether this had occurred during the past year, was endorsed by a total of 11% (although logically, all the latter individuals theoretically also should have endorsed the first item). When collapsing these two items into one variable, a total of 12% endorsed a history of past-year or previous over-indebtedness. For lifetime history of over-indebtedness, this was clearly more common with increasing levels of gambling problems; 3%, 9%, 13%, and 46% of respondents with no risk gambling, low-risk gambling, moderate-risk gambling, and problem gambling, respectively (p < 0.001, chi-squared, linear-by-linear). Over-indebtedness was reported by 28% of those reporting past 30-day online casino gambling and sports live betting, 18% of recent online casino gamblers (without sports live betting), and 6% in recent live sports bettors (without online casino) and 6% in those reporting none of these two gambling activities, respectively. Comparisons between respondents with and without a history of over-indebtedness are reported in **Table 4**.

**Table 4:** Unadjusted comparison of respondents with and without a history of over-indebtedness, respectively (N = 1,004). Chi-squared analysis and Mann-Whitney comparison.

	Over-indebtedness, % (n)	No over-indebtedness, % (n)	p value
Male gender	63 (75)	80 (711)	<0.001
Age group			<0.001
- 18–24	7 (8)	4 (33)	
- 25–29	12 (14)	7 (65)	
- 30–39	33 (40)	20 (180)	
- 40–49	26 (31)	22 (196)	
- 50–59	13 (16)	23 (203)	
- 60–69	7 (8)	15 (133)	
- 70+	3 (3)	8 (74)	
Psychological distress	18 (IQR, 13.25–21.75)	9 (7–12)	<0.001
Alcohol problems	24 (29)	4 (36)	<0.001
Drug problems	13 (16)	2 (18)	<0.001
Tertiary education	43 (52)	47 (419)	0.40
Past 30-day money lost from gambling (Euros)***			<0.001
- <4.3	13 (15)	19 (171)	
- 4.3–8.7	10 (12)	10 (87)	
- 8.7–17.4	9 (11)	16 (138)	
- 17.4–34.8	13 (16)	17 (150)	
- 34.8–52.2	11 (13)	13 (118)	
- 52.2–87.0	11 (13)	9 (77)	
- 87.0–173.9	13 (16)	10 (86)	
- 173.9–434.8	8 (9)	4 (33)	
- 434.8–869.6	5 (6)	1 (11)	
- >869.6	8 (9)	1 (13)	
Past 30-day online gambling activity			< 0.001
- None	15 (18)	32 (280)	
- Online casino, no live betting	25 (30)	15 (134)	
- Live betting, no online casino	19 (23)	39 (342)	
- Both	41 (49)	14 (128)	

***Responses in local currency, and values expressed here in Euros (1 Euro corresponding to around 11.5 SEK).

In logistic regression, lifetime history of over-indebtedness was significantly associated with psychological distress, higher degree of problem gambling, and alcohol problems. When excluding the problem gambling (PGSI) in the second model, lifetime history of over-indebtedness was significantly associated with psychological distress, alcohol problems, and past 30-day combined online casino and live betting (**Table 5**).

**Table 5:** Variables associated with a history of over-indebtedness (N = 1,004). Hierarchical logistic regression, with or without problem gambling score (PGSI).

	Model 1 (including PGSI)*		Model 2 (excluding PGSI)*		
	OR	95% Confidence interval	OR	95% Confidence interval
Age group	1.03	0.86–1.23	0.93	0.78–1.09
Male gender	0.91	0.52–1.61	0.81	0.48–1.38
Tertiary education	0.65	0.40–1.05	1.72	0.45–1.14
Problem gambling score (PGSI, total)	1.15	1.09–1.21**	—	—
Psychological distress	1.17	1.12–1.23**	1.21	1.16–1.27**
Alcohol problems	2.40	1.17–4.91**	2.71	1.37–5.39**
Drug problems	1.25	0.48–3.30	1.56	0.64–3.80
Past 30-day money lost from gambling	0.92	0.83–1.03	1.04	0.95–1.15
Past 30-day online gambling activity (reference: neither online casino, nor sports live betting)				
- Online casino	1.52	0.72–3.22	1.89	0.93–3.85
- Sports live betting	0.98	0.48–1.99	0.90	0.45–1.81
- Online casino + sports live betting	1.57	0.74–2.29	2.18	1.08–4.43**

*Nagelkerke: 0.40 and 0.36.

**Significant association (p < 0.05).

### Expected Future Over-Indebtedness

Eight percent of respondents reported expected over-indebtedness for the next two months. This was reported by 20% of those reporting both online casino gambling and online sports betting, 15% of online casino gamblers, 4% of live sports bettors, and by 3% of those neither reporting online casino or online sports betting during the past 30 days. Comparisons between respondents with and without expected future over-indebtedness are reported in **Table 6**.

**Table 6:** Unadjusted comparison of respondents with and without expected future over-indebtedness, respectively (N = 1,004). Chi-squared analysis and Mann-Whitney comparison.

	Future over-indebtedness, % (n)	No future over-indebtedness, % (n)	p value
Male gender	20 (185)	41 (33)	<0.001
Age group			<0.001
- 18–24	9 (7)	4 (34)	
- 25–29	12 (10)	7 (69)	
- 30–39	37 (30)	21 (190)	
- 40–49	22 (18)	23 (209)	
- 50–59	14 (11)	23 (208)	
- 60–69	4 (3)	15 (138)	
- 70+	2 (2)	8 (75)	
Psychological distress	18 (IQR, 15–23)	9 (7–13)	<0.001
Alcohol problems	27 (22)	5 (43)	<0.001
Drug problems	17 (14)	2 (20)	<0.001
Tertiary education	38 (31)	48 (440)	0.10
Past 30-day money lost from gambling (Euros)***			<0.001
- <4.3	14 (11)	19 (175)	
- 4.3–8.7	6 (5)	10 (94)	
- 8.7–17.4	11 (9)	15 (140)	
- 17.4–34.8	12 (10)	17 (156)	
- 34.8–52.2	11 (9)	13 (122)	
- 52.2–87.0	14 (11)	9 (79)	
- 87.0–173.9	11 (9)	10 (93)	
- 173.9–434.8	6 (5)	4 (37)	
- 434.8–869.6	4 (3)	2 (14)	
- >869.6	11 (9)	1 (13)	
Past 30-day online gambling activity			<0.001
- None	11 (9)	31 (289)	
- Online casino, no live betting	30 (24)	15 (140)	
- Live betting, no online casino	16 (13)	38 (352)	
- Both	43 (35)	15 (142)	

***Responses in local currency, and values expressed here in Euros (1 Euro corresponding to around 11.5 SEK).

Expected future over-indebtedness, in logistic regression, was significantly associated with psychological distress, degree of problem gambling, and negatively associated with tertiary education. In the second model, when excluding the variable describing problem gambling (PGSI), future over-indebtedness was significantly associated with psychological distress, alcohol problems, negatively associated with tertiary education, and positively associated with online casino gambling (**Table 7**).

**Table 7:** Variables associated with expected future over-indebtedness (next 2 months, N = 1004). Hierarchical logistic regression, with or without problem gambling score (PGSI).

	Model 1 (incl PGSI)*		Model 2 (excl PGSI)*		
Age group	0.93	0.75–1.14	0.85	0.70–1.05
Male gender	1.02	0.53–1.96	0.88	0.47–1.64
Tertiary education	0.46	0.25–0.84**	0.53	0.30–0.93**
Problem gambling score (PGSI, total)	1.11	1.06–1.18**	—	—
Psychological distress	1.21	1.14–1.28**	1.24	1.18–1.31**
Alcohol problems	1.90	0.86–4.21	2.19	1.02–4.72**
Drug problems	1.80	0.67–4.87	2.15	0.84–5.49
Past 30-day money lost from gambling	0.93	0.81–1.05	1.03	0.92–1.16
Past 30-day online gambling activity (reference: neither online casino, nor sports live betting)				
- Online casino	2.42	0.96–6.12	2.75	1.10–6.87**
- Sports live betting	1.06	0.41–2.73	1.01	0.39–2.58
- Online casino + sports live betting	1.80	0.71–4.58	2.39	0.96–5.96

*Nagelkerke: 0.43 and 0.40.

**Significant association (p < 0.05).

### Loans, Debts, and Problem Gambling

Nine percent (n = 87) had a lifetime history of gambling-related borrowing. Within this group, 51% reported loans from private acquaintances, 41% from payday loans (“cash advances”), 34% unsecured bank loans, and 15% secured bank loans. In this group, 76% were problem gamblers and another 14% were moderate-risk gamblers. Among the 60 individuals reporting having borrowed money related to gambling during the past year, 88% were problem gamblers and 2% were moderate-risk gamblers. Fifty percent of respondent with problem gambling had ever borrowed money related to gambling, compared to 6% among moderate-risk gamblers, 2% of low-risk gamblers, and 1% of respondents with no risk. Having borrowed money related to gambling was reported by 27% of those reporting both online casino gambling and sports betting, 15% of recent online casino gamblers, 2% among live bettors, and by 2% of those reporting neither of these two gambling activities during the past 30 days.

Debts sent to the enforcement authority during the past year were reported by 8% of respondents (16% among those reporting both online casino and sports betting, 13% in recent online casino gamblers, 4% of sports live bettors, and 4% of those reporting neither of these two recent gambling activities).

## Discussion

The present study examined over-indebtedness and problem gambling in online gamblers, in a sample defined by the reporting of having gambled on either online casino or sports betting, the two gambling activities reported to be the most common in the gambling disorder treatment setting, on at least ten occasions during the past year. Altogether, hypotheses were confirmed; problem gambling and over-indebtedness, as hypothesized, differed markedly across types of gambling activities, with associations partly remaining when controlling for co-variates. Likewise, as hypothesized, no gender differences were seen with respect to problem gambling or over-indebtedness in the adjusted analyses. The main findings demonstrate a very high prevalence of problem gambling in individuals gambling in these gambling activities, and very high rates of over-indebtedness, with large differences depending on the recent gambling activities reported. Rates of problem gambling and over-indebtedness were markedly higher in individuals reporting recent online casino gambling, alone or in combination with online sports betting. In addition to the gambling pattern, over-indebtedness was associated with psychological distress and alcohol problems, and, for expected future over-indebtedness, with a lower level of schooling. As hypothesized, and contrary to previous literature in problem gambling overall, in the present study of online gamblers, problem gambling, and over-indebtedness were not associated with male gender in the adjusted models. Also, in an unadjusted analysis, problem gambling was more than twice as common in women than in men.

### High Degree of Problem Gambling in Online Gamblers

The present study demonstrated a high prevalence of past-year problem gambling in web panel participants who endorsed the criteria of being online gamblers during the past year. Thus, the very high prevalence of PGSI-measured problem gambling and moderate-risk gambling confirm previous hypotheses of a particularly problematic picture in online gamblers (14–17, 36). Specifically, problem gambling has been described to be more common in online gamblers than in offline gamblers (37), and inherent characteristics of the online gambling modality may inhibit self-control during gambling (38). The fact that gambling is carried out online may not in itself cause gambling problems, but the online modality may increase the risk in some individuals (39). Previous reports have suggested, for example, that the speed of gambling and the high degree of availability may contribute to more pronounced problems in online gambling (14, 40).

### The Role of Online Casino in Problem Gambling in Online Gamblers

In the present study, online casino stands out as being closely related to higher rates of problem gambling and over-indebtedness. The unadjusted percentage who fulfilled either problem gambling or moderate-risk gambling was more than three times higher in recent online casino gamblers than in those neither online casino nor sports live betting during the past month, and even higher in those reporting both online casino and live sports betting. These findings are in line with clinical data reporting that online casino represents the most common gambling activity in patients seeking treatment for a gambling disorder, and also, online sports betting is one of the most common gambling activities in that context (18). The associations between specific online gambling activities and problem gambling and over-indebtedness in the present study remained when controlling for past 30-day gambling losses, a variable included as an overall measure of gambling involvement. Thus, although subjects reporting a recent history of online casino gambling had particularly high problem rates, the actual gambling problem could theoretically be due also to a different, land-based gambling activity. However, if so, nothing in the present study indicates that the overall extent of gambling losses would be the sole explanation of problem gambling and over-indebtedness, but in each statistical model, important associations with specific online gambling patterns remained. Also, the present study addressed online gamblers specifically, in light of the potential hazards of online gambling, and it is therefore beyond the scope of the present study to assess potentially specific risks of problem gambling in separate land-based gambling activities.

While the present study demonstrates a high likelihood of problem gambling in online gamblers, it theoretically cannot be excluded that problems are instead caused by a concurrent pattern of another gambling activity than the two activities (online casino gambling and live sports betting) analyzed as a focus of this paper. In the sub-group of respondents reporting neither of these gambling activities during the past 30 days, however, non-live sports betting was actually less common in the group reporting neither online casino nor live sports betting during the past 30 days (56%) than in the live betting group (76%, p < 0.001) and the combined online casino and live betting group (68%, p = 0.01). Also, in the group without recent online casino or live sports betting during the past 30 days, online horse betting was no more common than in recent live bettors or online casino gamblers, and in this group, land-based casino gambling and land-based electronic gambling machines were rare (3% and 1%, respectively). Thus, this group reporting neither online casino nor live betting appears to have a less intense gambling pattern, and along with the live betting only group, this group has clearly lower rates of problem gambling and over-indebtedness compared to recent online casino gamblers.

Based on the results of the present study, it can be discussed why online casino contributes to this extent to gambling problems. Online casino is known to be the gambling modality most commonly promoted in gambling advertising in Sweden (24). However, there is not a vast literature addressing specific risks of online casino gambling, over and above the literature describing the potentially harmful components in online gambling in general (39) and the higher prevalence of problem gambling in online gambling overall (37). A recent study, which demonstrated the links between specific gambling modalities and PGSI scores, reported a significant association between problem gambling and the use of electronic gambling machines online (41), which corresponds to the pattern of online casino gambling primarily promoted in Swedish media. This is suspected to represent a large part of the gambling referred to as online casino gambling in the present study. Clearly, given the relative novelty of research in separate gambling activities happening primarily online, more research is needed in order to elucidate the rates of problematic and non-problematic gambling patterns in online casino gambling. Here, however, given the large differences in problem gambling and over-indebtedness in online gamblers depending on whether past-month online casino gambling was reported or not, lends early support to this gambling pattern as particularly harmful.

### High Degree of Problem Gambling and Over-Indebtedness in Female Online Gamblers

One of the clearest findings of the present paper was the lack of association between male gender and problem gambling, or over-indebtedness, which otherwise would be expected, based on studies showing that men are overrepresented in problem gambling, both in the general population (19) and in clinical settings (18). In the present study of online gamblers, the large majority of respondents were men, with a male-to-female ratio very similar to what is seen in clinical studies of gambling disorder patients in Sweden (18, 42). Despite this male predominance of subjects included because of online gambling, in the unadjusted analyses of the present study, female respondents were markedly more likely to fulfil criteria of problem gambling. While this may seem surprising in comparison to gamblers or to the general population in general, the lack of an association with male gender – and even the overrepresentation of women among problem gamblers in the unadjusted model – may be due to specific characteristics of the specific population studied here. What characterizes the present study population is their history of having gambled online at least ten times during the past year. It previously has been shown from Sweden that women who seek treatment for a gambling disorder are more likely to report online casino gambling than their male counterparts, who were – in that study – more likely to report sports betting (18). Also, a recent Swedish public health report (41) revealed a rapidly increasing percentage of women among those with the most severe level of problem gambling (25).

Some previous research has suggested that debts related to problem gambling tend to be lower in women than in men (43). However, in contrast, a study in US helpline callers identified higher financial problems and gambling-related borrowing in women than in men (44), thus more consistent with the findings of the present study. The present study suggests the need to increase attention to gambling as a mediator of over-indebtedness in women, at least on a level comparable to their male counterparts, as the adjusted analysis failed to demonstrate any gender difference. Meanwhile, the unadjusted analysis rather calls for increased attention particularly to female gambling. It can be concluded from the present study that on the online gambling market, with a large market share of online casino, gender distribution may be different from what is typically known in the research are of problem gambling. Psychiatric comorbidity is known to be more common in women with a gambling disorder than among their male counterparts (18, 42, 45, 46), and suicidal ideation among patients seeking treatment for a gambling disorder is more common in women than in men (7). For this reason, the exposure to gambling may affect men and women differently, and it cannot be excluded that the increase in online gambling during the past decade makes high-risk gambling available to groups with a traditionally lower prevalence of problem gambling, particularly women. If so, such an explanation would indicate a novel recruitment of new groups to the newer gambling activities online. Here, more research is needed in order to further elucidate whether the previously unseen gender proportions are repeated in other studies, and which factors mediate these associations. In particular, there is need for research addressing the risks of problem gambling in women, and public health efforts in the area may need to address women specifically.

### High Level of Over-Indebtedness in Online Gamblers

Level of education was a protective factor with respect to over-indebtedness in the adjusted analyses, although paradoxically, the association between tertiary education and problem gambling went in the opposite direction. Here, a higher level of education was associated with a higher likelihood of problem gambling. The latter is in contrast with previous research pointing out lower education as a risk factor of problem gambling [e.g. (4, 47)]. While some previous data indicated a protective role of higher education vis-à-vis debts (48), studies examining associations between the level of education and debts have presented somewhat ambiguous results (49). In the present study, it cannot be excluded that the surprising finding of a positive association between higher education and problem gambling may be due to characteristics related to the present population being online gamblers, specifically.

Over-indebtedness is reported to occur in between 6% and 9% of the general population, also using a subjective definition (35). Thus, the percentage reporting over-indebtedness in the present study was only moderately elevated compared to that figure, although with large differences across reported gambling activities. Indeed, over-indebtedness reached a level three to four times higher in the group reporting both online casino and live sports betting, compared to the general population.

A very high percentage of those reporting debts to the enforcement authority also reported recent gambling on gambling activities typically perceived as risky. However, it is remarkable that a very high percentage of these respondents had a highly risky gambling pattern during the past month, as the debts furthered to the enforcement authority are unlikely to be related to past-month gambling. Thus, the recent gambling involvement described is likely to have occurred after—and in spite of—these individuals’ over-indebtedness. This finding may have implication for the risk assessment and need for effective screening and preventive work in gambling situations, where it would be reasonable to provide specific prevention tools or advice for the group of online gamblers who enter a gambling situation despite an ongoing involvement with enforcement authorities or in a similar situation of over-indebtedness. Likewise, for enforcement authorities and for other organizations involved in prevention, assessment or interventions targeting financial debts, the risk of an ongoing problematic gambling pattern likely needs assessment. In line with previous research suggesting problem gambling screening by consumer credit counsellors or by financial institutes (50, 51), the present study points to the need to identify problematic gambling patterns also in such settings outside of traditional treatment or counseling settings.

### Study Implications

The high rates of problem gambling and over-indebtedness in the present study call for preventive and policy efforts related to the increasing proportion of online gambling harm. Also, given the differences in harm demonstrated across different gambling activities, preventive measures may need to address online casino gambling in particular. Importantly, this calls for regulating legislation regarding online casino gambling. This could include the regulation of the content or extent of online casino advertising in television and in other media, where it has been shown that this particular gambling activity is predominating (24). Also, the present findings call for responsible gambling interventions aiming to control or discontinue gambling in online gamblers showing signs of addictive or problematic behavior. This could include available and easy-to-access self-exclusion tools in online gamblers, but may also include motivational interventions with clients in close association with major losses or in periods of uncontrolled gambling. Here, given the inherent commercial value of large-scale gambling to a commercial gambling operator, strict legal regulations are likely to be needed.

Likewise, in the health care sector, the present findings indicate the need for increased attention to online gambling as a potential hazard to health, with or without parallel risk factors such as psychological distress. Thus, active screening for online gambling may be warranted in people treated for poor mental health. Also, given the connection between online gambling – and particularly certain gambling activities – and over-indebtedness in the present study, authorities who meet individuals with debts and financial difficulties, such as social authorities or enforcement authorities, may need to address online gambling in screening tools. Clearly, over and above well-known correlates such as poor mental health, certain gambling activities stand out as more clearly associated to problem gambling than others, and may require particular attention in screening tools and assessment interviews in clinical settings. The same may apply to consumer credit counselors and to collection and enforcement authorities, where the active screening for online gambling may improve the understanding of the background of debts.

For the field of problem gambling research, the findings of the present study call for future study designs which allow for the study of causal associations of specific gambling patterns and gambling-related harm. For example, a longitudinal study of individuals initiating online casino gambling, live betting or other gambling activities would be of great value. Also, it would add to the present field if such study designs can include objective outcome measures, such as bank account data or other absolute descriptions of gambling-related harm, in addition to more traditional self-report measures.

### Limitations

The present study has a number of limitations. The study is a cross-sectional study where the longitudinal course of gambling patterns and financial debts cannot be elucidated, and the study is also limited by its design as a web-based self-report study. The cross-sectional study design, for example, limits the capacity of the study to conclude whether psychological distress, associated with both problem gambling and over-indebtedness in the study, is caused by these outcomes or instead contributes to it. The measure of psychological distress, the Kessler-6, has been internationally tested and validated with satisfactory results, but has not been validated specifically in Swedish. Also, gambling patterns were described only for the past 30 days, in order to assess gambling patterns as closely to the date of assessment as possibly. This past 30-day measure is likely to have relevance for the current estimate of problem gambling and for the measure of expected future over-indebtedness. In addition, recent gambling patterns may have implications for individuals who are already in severe financial debts and for whom a past 30-day gambling pattern may be considered harmful. Meanwhile, of course, predictions cannot be made about how the recent gambling pattern affects historic data on over-indebtedness, which is a limitation, and which would be studied better in a longitudinal study design. In addition, generalizability cannot be fully established, although the present study addresses the general population, it could be shown that the web panel members recruited to the present study had a higher level of schooling than the general population of Sweden. In the general population aged 16 to 74 years, 35% have tertiary education, compared to the 47% of the present study (52). It cannot be excluded that this is due to the data collection being carried out in a pre-existing web panel, as people who engage in a web survey panel addressing gambling may potentially have a different socio-demographic characteristic than others. Based on previous knowledge, the higher level of education would theoretically underestimate problem gambling rather than the opposite; thus, this potential selection bias is at least unlikely to have reported too high estimates of gambling problems. This assumption is however in some contrast with the present study finding that problem gamblers actually had higher education than non-problem gamblers. It remains to be studied in future research whether the risk of harm in online gambling may demonstrate an opposite association to level of schooling than what has been seen traditionally.

In the analyses of over-indebtedness, due to the survey-based and cross-sectional design of the study, several potential correlates of debt have not been controlled for. Although somewhat beyond the aims of the present study, theoretically, it would be of value to study such factors as living conditions, socio-economic variables, and deprivation. However, level of education was surprisingly positively associated with problem gambling in the present sample but negatively associated with over-indebtedness. Future research may need to study further measures of socio-demographics and socio-economy, in order to more fully outline the associations between separate gambling activities and the development of debts.

In the present study, the study sample was defined based on past-year online gambling, focusing on online casino and online betting, but analyses of the correlates of gambling consequences focused specifically on online casino gambling and live sports betting. These definitions were based on documented clinical experience of online casino and sports betting as the predominating gambling activities reported by those seeking treatment (18, 19). While this is potentially a limitation, a sensitivity analysis was carried out in order to elaborate on the role of online horse betting in the present context. However, when including this past 30-day gambling activity, problem gambling was as low as 1% in those reporting this gambling activity specifically (and neither online casino nor live sports betting), and none of the combinations with online horse betting was associated with higher rates of problem gambling. For these reasons, horse betting online during the past 30 days was not considered for further analyses in the study. In addition, one limitation is that prior to the study, a power analysis could not be calculated, as previous studies did not allow for the prediction of potential outcomes in different groups in this sample of online gamblers. Instead, available population data indicated the prevalence of problem gambling across gambling activities but without the separation of different gambling modalities; for example, problem gambling in any casino gambling had previously been reported in public health report data, without the separation of land-based and online casino gambling (53). Finally, another limitation of the paper may be the fact that the study did not control for multiple comparisons, given the aim to study both problem gambling, previous over-indebtedness and future over-indebtedness. Here, it will be of importance for future studies in online gamblers to replicate and corroborate the present findings.

## Conclusions

The present study demonstrates that in online gamblers, in the Swedish setting with its predominance of online casino and sports betting in treatment-seeking patients, the gender distribution is different from that expected from the literature; female gender was more common in problem gamblers and in gamblers with over-indebtedness, and even when controlling for a number of potential risk factors and for overall past-month gambling losses, male gender was not—as traditionally seen—associated with problem gambling or over-indebtedness. Remarkably elevated rates of problem gambling and over-indebtedness were seen in recent online casino gamblers (with or without concurrent live sports betting). The findings call for preventive efforts addressing online gambling-related harm, and with a particular emphasis on online casino gambling. More remains to be understood about the role of gender in gambling, and the present findings call for further study of female online gamblers, and further clinical attention to the risk of problem gambling in women with novel gambling patterns.

## Data Availability Statement

The datasets generated for this study are available on request to the corresponding author.

## Ethics Statement

The studies involving human participants were reviewed and approved by Regional ethics board Lund, Sweden. The patients/participants provided their written informed consent to participate in this study.

## Author Contributions

AH was the principal investigator of the study, and carried out statistical analyses and wrote the draft of the paper. CW was the co-investigator of the study and made large contributions to the interpretation of the data and to the editing of the paper.

## Funding

The present study was financed by a study-specific grant from the research council of the Swedish Enforcement Authority. In addition, the first author holds a position at Lund University which is sponsored by the Swedish government-owned gambling operator Svenska spel AB, as part of that company’s responsible gambling strategy, and the first and the second author receive study-specific and non-specific research funding from Svenska spel AB and from the research council of Svenska spel AB. Svenska spel AB had no role in the present study. The Swedish Enforcement Authority had no role in the interpretation of data or in the writing of the study report.

## Conflict of Interest

The authors declare that the research was conducted in the absence of any commercial or financial relationships that could be construed as a potential conflict of interest.
